# Temporal association of diffuse large B-cell lymphoma with PD-1 inhibitor therapy in a patient with gastric adenocarcinoma: a case report

**DOI:** 10.3389/fimmu.2026.1763711

**Published:** 2026-02-12

**Authors:** Yiqing Jiang, Kun Wu, Jia Hu, Mingxin Yu, Yuhang Xu, Zihan Xu, Rongxuan Cao, Yi Zhang, Yanfang Gao, Shuzhen Liu, Yanhong Ding

**Affiliations:** 1 Weifang People’s Hospital, Shandong Second Medical University, Weifang, China; 2 Department of Respiratory and Critical Care Medicine, Sunshine Fusion Hospital, Weifang, Shandong, China; 3 Hematology of the First Affiliated Hospital, WeiFang People’s Hospital, Shandong Second Medical University, Weifang, China; 4 Oncology Laboratory of the First Affiliated Hospital, WeiFang People’s Hospital, Shandong Second Medical University, Weifang, China

**Keywords:** diffuse large B-cell lymphoma (DLBCL), immune checkpoint inhibitors (ICIs), immune-related adverse events (irAEs), PD-1 inhibitors, sintilimab

## Abstract

**Background:**

Immune checkpoint inhibitors (ICIs) have demonstrated substantial clinical benefit across a wide range of malignancies. With their expanding use, uncommon immune-related events, including hematologic abnormalities and lymphoid proliferations, are increasingly recognized. However, a causal relationship between ICI exposure and lymphoma development remains unproven.

**Case description:**

We report a 70-year-old woman with moderately to poorly differentiated gastric adenocarcinoma who was diagnosed with diffuse large B-cell lymphoma (DLBCL) during postoperative treatment with FOLFOX chemotherapy combined with the PD-1 inhibitor sintilimab. During therapy, the patient developed recurrent pleural and pericardial effusions. Early pleural fluid cytology revealed atypical lymphoid cells with occasional Epstein–Barr virus–encoded RNA (EBER) positivity, but immunophenotypic and clonality assessments were not performed, precluding a definitive lymphoma diagnosis at that time. Subsequent cytological, immunophenotypic, and molecular studies confirmed Ann Arbor stage IV DLBCL, predominantly presenting as malignant effusions. The patient achieved remission with R-CHOP therapy but later experienced relapsed and refractory disease.

**Conclusion:**

This case illustrates a temporal association between PD-1 inhibitor–based therapy and the diagnosis of DLBCL. Given the lack of baseline systemic staging, overlapping PET/CT findings, early diagnostic uncertainty in effusion cytology, and potential contributions from chemotherapy-induced immune perturbation and EBV-associated processes, a direct causal relationship cannot be established. This report underscores the importance of comprehensive baseline evaluation and cautious interpretation of atypical lymphoid findings during immunotherapy.

## Introduction

Immune checkpoint inhibitors (ICIs) have revolutionized cancer therapy by enhancing antitumor immunity. Their primary mechanism involves the blockade of the PD-1/PD-L1 axis, which restores T-cell activation and improves immune recognition and elimination of tumor cells (1). This mechanism has positioned immunotherapy as a promising approach for achieving durable disease control and, in selected settings, may contribute to long-term remission (2, 3).

With the widespread clinical use of ICIs, immune-related adverse events (irAEs) are increasingly recognized, with severity ranging from mild symptoms to life-threatening conditions. Although secondary lymphomas appear to be rare, their potential severity warrants close clinical monitoring. The 2025 Drug Vigilance Summary in the Journal for ImmunoTherapy of Cancer reviewed lymphoma cases reported after ICI exposure and identified a signal involving secondary pulmonary lymphoma (SPL), including invasive B-cell lymphoma. While such pharmacovigilance signals do not establish causality, they underscore the importance of clinical vigilance, particularly when new lymphadenopathy or atypical effusions emerge during or after immunotherapy.

Clinically, diffuse large B-cell lymphoma (DLBCL) often presents with lymphadenopathy or extranodal involvement, which may be misinterpreted as cancer progression, thus posing challenges for early diagnosis and potentially delaying appropriate treatment. The management of such cases may be further complicated by the interruption of immunotherapy due to severe irAEs, which could impact tumor control and influence recurrence risk (4). It is crucial to recognize that these phenomena may be due to an immune dysregulation process rather than direct causality from the immunotherapy itself. This necessitates a cautious approach in the interpretation of new lymphoid findings and highlights the importance of maintaining a high index of suspicion when managing cancer patients undergoing ICI therapy.

## Case presentation

**Figure 1:**
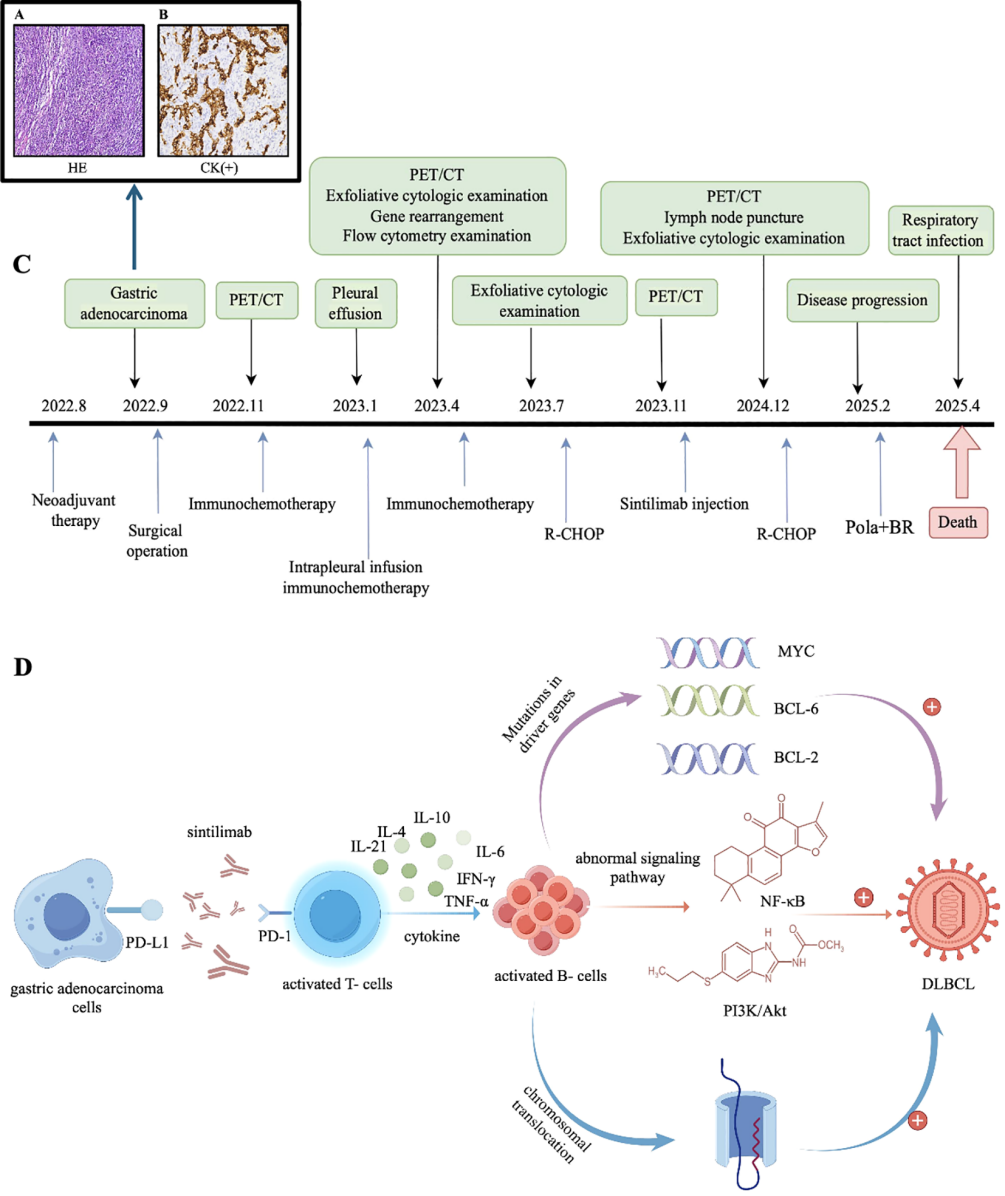
Postoperative pathological findings, treatment timeline, and schematic representation of underlying mechanisms. **(A)** Total gastrectomy specimen demonstrating poorly differentiated adenocarcinoma. **(B)** Tumor cells exhibit diffuse pan-cytokeratin (CK) positivity on immunohistochemistry, indicative of epithelial differentiation. **(C)** The timeline of therapeutic interventions administered to this patient. **(D)** Three hypothetical mechanisms by which PD-1 blockade, via modulation of T-cell function, may promote aberrant B-cell proliferation and ultimately contribute to the development of diffuse large B-cell lymphoma (DLBCL). (This schematic illustrates hypothetical mechanisms and does not imply causality).

A 70-year-old woman presented with a two-month history of mild abdominal discomfort. In August 2022, upper gastrointestinal endoscopy performed at Weifang Sunshine United Hospital revealed mass lesions at the gastroesophageal junction and gastric fundus. Histopathological examination of biopsy specimens confirmed poorly differentiated gastric cardia adenocarcinoma, and Epstein–Barr virus–encoded RNA (EBER) *in situ* hybridization was negative. The patient was transferred on August 14, 2022, to Weifang People’s Hospital for pathological review, which confirmed moderately to poorly differentiated gastric adenocarcinoma. She subsequently received two cycles of neoadjuvant FOLFOX chemotherapy (oxaliplatin, leucovorin, and 5-fluorouracil).Preoperative evaluation in September 2022 showed negative HIV serology; human herpesvirus 8 (HHV-8) testing was not performed. The patient underwent laparoscopic total gastrectomy with regional lymph node dissection and Roux-en-Y reconstruction. Postoperative histopathology demonstrated diffuse poorly differentiated gastric adenocarcinoma with prominent lymphocytic infiltration and no lymph node metastasis (**Figures 1A, B**).

**Figure 2:**
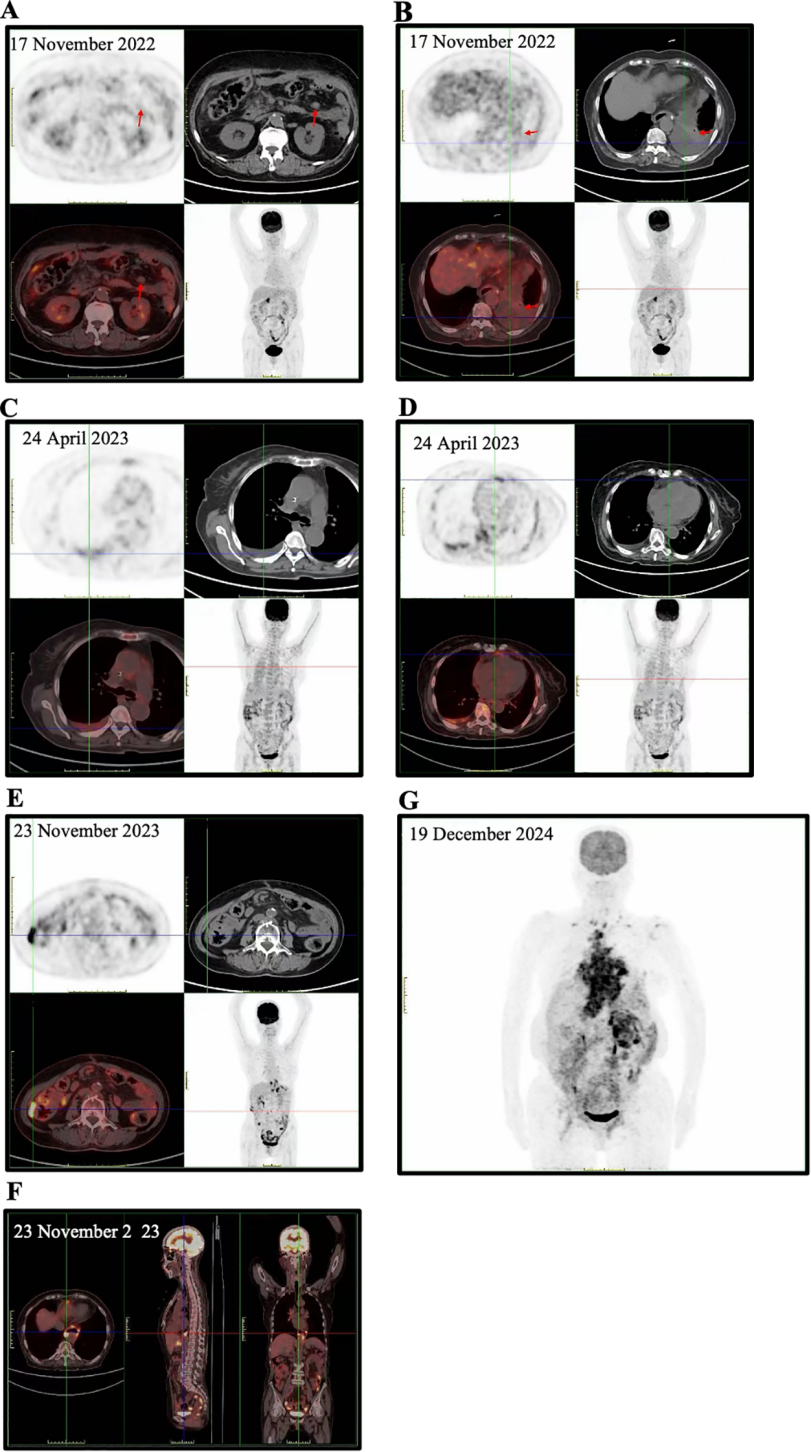
The results of four imaging examinations that documented changes in the patient’s condition. **(A)** PET/CT demonstrates a hypermetabolic nodule in the left abdominal cavity. **(B)** PET/CT reveals left-sided pleural effusion. **(C–D)** PET/CT shows increased metabolic activity within pleural and pericardial effusions. **(E-F)** PET/CT demonstrates newly identified heterogeneous thickening of the right colonic mesentery with increased FDG uptake, along with newly developed abnormal hypermetabolism at the distal esophageal anastomotic site. **(G)** PET/CT reveals multiple hypermetabolic lesions distributed throughout the body.

In November 2022, postoperative surveillance positron emission tomography/computed tomography (PET/CT) revealed a hypermetabolic nodule in the left abdominal cavity (**Figure 2A**) and left-sided pleural effusion (**Figure 2B**), raising suspicion for metastatic lymphadenopathy. From November to December 2022, the patient initiated adjuvant therapy with two cycles of FOLFOX combined with the programmed cell death protein 1 (PD-1) inhibitor sintilimab. In January 2023, diagnostic thoracentesis demonstrated abundant, diffusely distributed, relatively monomorphic atypical lymphoid cells. EBER *in situ* hybridization showed focal positivity. Given that exfoliative cytology alone was insufficient for definitive diagnosis, B-cell receptor gene rearrangement analysis was performed and revealed monoclonal B-cell rearrangement (**Table 1**). After multidisciplinary pathological consultation and consideration of prior cytotoxic and immunomodulatory therapy, the findings were interpreted as treatment-associated abnormal B-cell proliferation. Continued antitumor therapy with close surveillance was recommended. Between January and early April 2023, the patient received two intrapleural treatments with reduction of pleural effusion and subsequently completed six additional cycles of FOLFOX combined with sintilimab.

**Table 1:** The analysis of B-cell receptor gene rearrangements.

Test item	Test content	Test target	Test result
B-cell receptor gene Rearrangement	IGH Tube A	FR1-JH	Monoclonal Proliferation
	IGH Tube B	FR2-JH	Polyclonal Proliferation
	IGH Tube C	FR3-JH	Polyclonal Proliferation
	IGH Tube D	DH-JH	Polyclonal Proliferation
	IGH Tube E	DH7-JH	Polyclonal Proliferation
	IGL Tube	Vλ-Jλ	Polyclonal Proliferation
	IGK Tube A	Vκ-Jκ	Polyclonal Proliferation
	IGK Tube B	Vκ-Kde+intron-Kde	Polyclonal Proliferation
		Control Gene Tube	400bp

Polyclonal proliferation across multiple regions may indicate reactive B-cell expansion, commonly observed in normal immune responses. However, the presence of monoclonal proliferation in specific regions suggests clonal B-cell expansion, which is typically associated with malignant lymphoproliferative disorders, such as diffuse large B-cell lymphoma (DLBCL). (Test specimen: pleural effusion. Testing method: capillary electrophoresis-based genetic analysis. Tumor cell content (%): 70).

**Figure 3:**
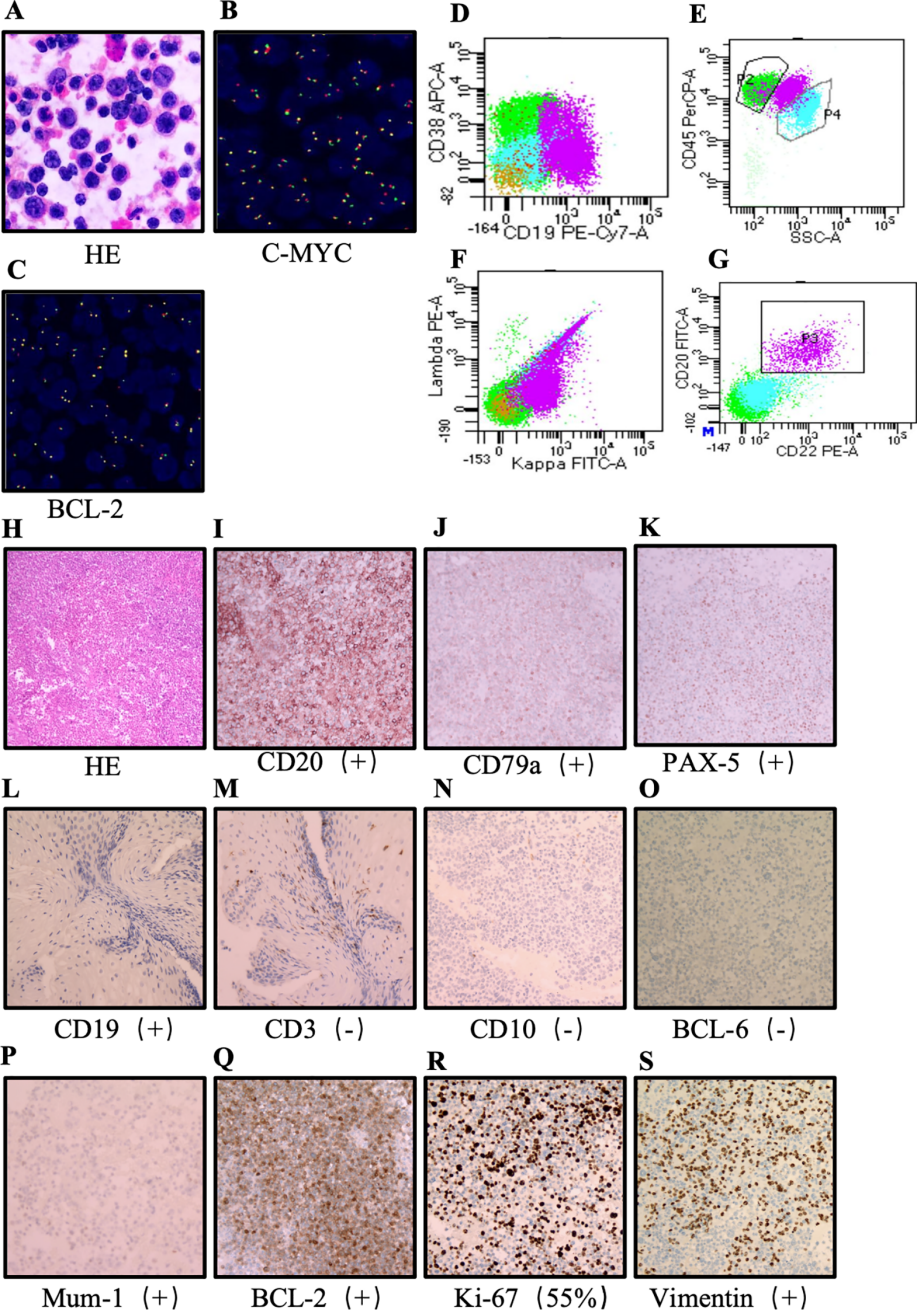
Pathological examination of pleural effusion, FISH analysis, and flow cytometry; comprehensive analysis of pericardial effusion pathology and immunohistochemical findings. **(A)** Both pleural effusion smears and cell blocks demonstrated a significant proliferation of atypical lymphocytes. **(B)** No breakage was observed in the C-MYC gene; however, 57% of cells exhibited C-MYC gene amplification. **(C)** No breakage was observed in the BCL-2 gene; 13% of cells showed amplification of the BCL-2 gene. **(D)** The abnormal cell population was identified as B-cells. **(E)** The purple cell population represents the abnormal cells, which were notably larger than the normal cells, indicating that the abnormal cells are of a large-cell phenotype. **(F)** The purple cell population exclusively expressed Kappa light chains and not Lambda light chains, confirming that the abnormal clone is monoclonal. **(G)** The expression of CD20 and CD22, along with the monoclonality observed in panel F, further substantiates that the abnormal cells are mature. **(H)** Both smears and cell blocks from the pericardial effusion exhibited a large number of morphologically consistent atypical lymphocytes. **(I)** Diffuse strong positivity for CD20 is a hallmark of B-cells. **(G)** CD79a positivity serves as a marker for B-cell membrane and cytoplasm. **(K)** Nuclear positivity for PAX5, a highly specific B-cell transcription factor, was observed. **(L)** CD19 positivity further supports the B-cell origin. **(M)** CD3 negativity rules out T-cell lymphoma. **(N-P)** Negative for CD10, BCL-6, and positive for Mum-1, collectively indicating a non-germinal center B-cell subtype. **(Q)** BCL-2 is negativity. **(R)** Ki-67 was expressed in 55% of cells, indicating a high proliferative index and suggesting a high degree of malignancy. **(S)** Vimentin is positivity.

In April 2023, follow-up ultrasonography identified a large right-sided pleural effusion. Repeat cytological analysis (**Figures 3A–C**), flow cytometry (**Figures 3D–G**), and external expert consultation favored a diagnosis of diffuse large B-cell lymphoma (DLBCL). PET/CT showed resolution of metabolic activity in the previously noted left abdominal lesion but increased metabolic activity in pleural and pericardial effusions (**Figures 2C, D**), consistent with malignant effusions. After one additional cycle of the original regimen, the patient was evaluated at Peking University Cancer Hospital on April 28, 2023. Multidisciplinary consultation raised the possibility of concomitant gastric adenocarcinoma and DLBCL; however, repeat tissue biopsy was recommended but not obtained, and the nature of the lymphoid proliferation—reactive, immune-related, or overt lymphoma—remained uncertain. In May 2023, the patient returned to Weifang People’s Hospital. Given the absence of significant metabolic activity in abdominal lymph nodes, resolution of bilateral pleural effusions, and apparent clinical stability of the lymphoproliferative process, she received two cycles of sintilimab maintenance therapy between May and June 2023 following comprehensive reassessment.

In July 2023, the patient was readmitted with chest tightness and dyspnea. Imaging revealed recurrent pleural and pericardial effusions. Pericardial fluid cytology demonstrated numerous morphologically uniform atypical lymphoid cells (**Figure 3H**). Integrating immunohistochemical findings (**Figures 3I–S**) with longitudinal clinical data, a definitive diagnosis of DLBCL was established. According to the Ann Arbor staging system, the disease was classified as stage III, with elevated lactate dehydrogenase (341 U/L). From July to November 2023, the patient received six cycles of R-CHOP chemotherapy. Post-treatment PET/CT demonstrated complete resolution of pleural, pericardial, and pelvic effusions. However, new hypermetabolic lesions were detected in the right colon (**Figure 2E**) and distal esophageal anastomosis (**Figure 2F**), raising concern for recurrent or metastatic gastric adenocarcinoma. The patient continued sintilimab maintenance therapy until February 2024 and received two cycles of oral tegafur, which was discontinued due to severe gastrointestinal toxicity.

**Figure 4:**
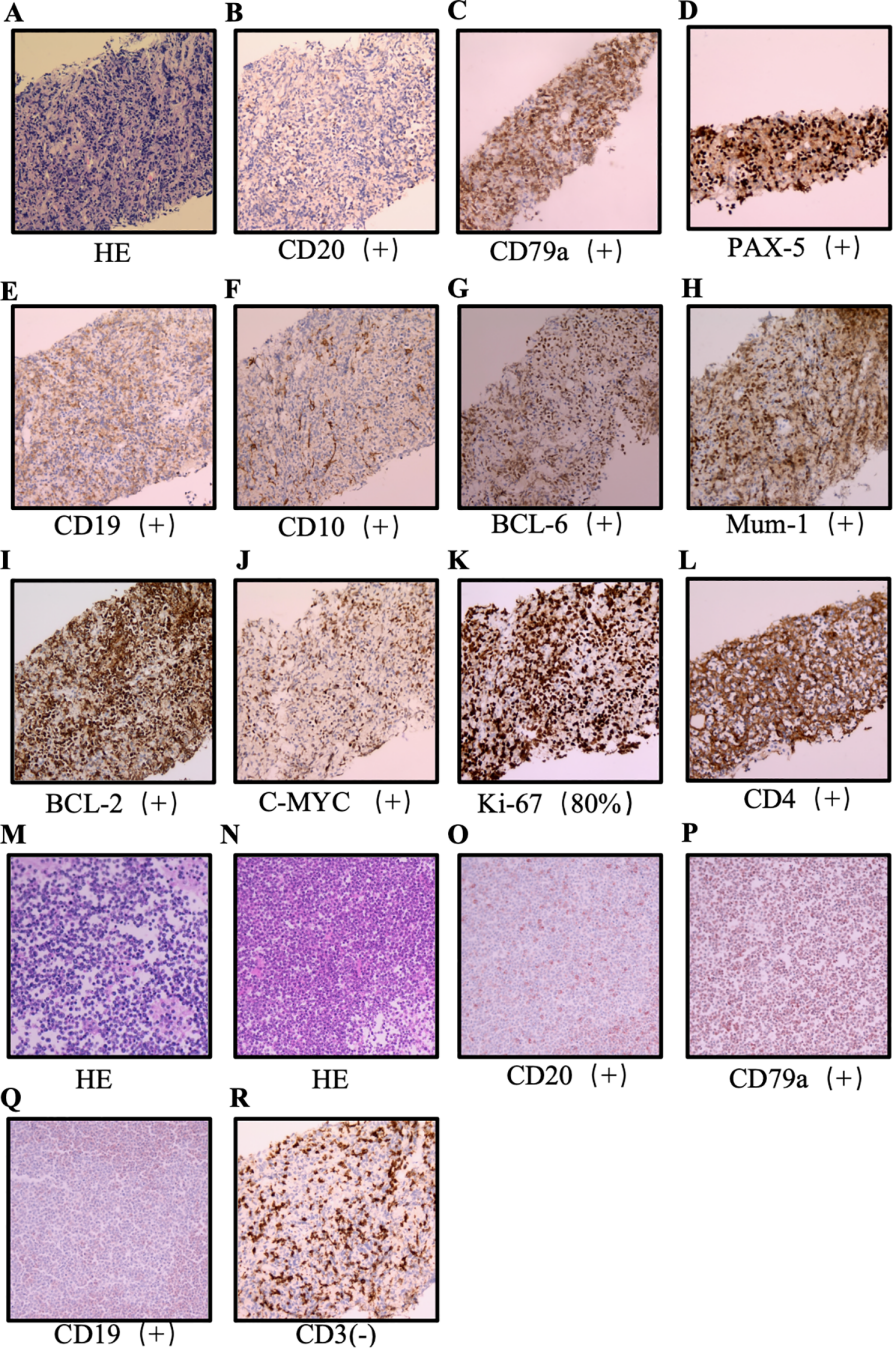
Pathological Findings from the Retroperitoneal Lymph Node Biopsy and Analysis of Pleural and Abdominal Effusions in the Patient. **(A)** Retroperitoneal lymph node biopsy reveals lymphoid hyperplasia. **(B–E)** CD20 shows rare positivity, CD79a is positive, PAX5 is positive in the nucleus, and CD19 shows partial positivity, collectively indicating that the abnormal cells are B cells. **(F–H)** CD10 shows slight positivity, BCL-6 is partially positive, and 75% of the cells are positive for Mum-1, indicating a non-germinal center B cell subtype. **(I, J)** BCL-2 is positive in 80% of cells and C-MYC is positive in 65% of cells. **(K)** Ki-67 expression is 80%, indicating high proliferation and a high degree of malignancy. **(L)** Vimentin is positive. **(M)** Pleural fluid smear and cell block show numerous atypical lymphoid cells with consistent size and shape. **(N)** Peritoneal fluid smear and cell block demonstrate tumor cells. **(O–R)** CD20 shows diffuse strong positivity, CD79a is positive, CD19 is positive, and CD3 is negative, further confirming the abnormal cells as B lymphocytes.

In November 2024, the patient was rehospitalized with bilateral lower extremity edema and progressive dysphagia. In December 2024, biopsies of retroperitoneal lymph nodes and ascitic fluid were performed. Lymph node histopathology demonstrated lymphoid proliferation with broad immunophenotypic positivity (**Figures 4A–L**). Ascitic fluid cytology confirmed malignant lymphoid cells (**Figures 4M–Q**), and PET/CT revealed disseminated hypermetabolic lesions (**Figure 2G**). DLBCL demonstrated a double-expressor phenotype by immunohistochemistry, with MYC and BCL2 overexpressed in ~65% and ~80% of tumor cells, respectively; FISH for MYC, BCL2, and BCL6 rearrangements was not performed. The patient received one cycle of R-CHOP chemotherapy.

In January 2025, the patient was readmitted due to disease progression. Although a repeat biopsy was recommended, the family declined; the patient subsequently received an additional cycle of R-CHOP chemotherapy before discharge. On January 20, 2025, the patient was hospitalized again for treatment-related myelosuppression—manifested primarily as leukopenia and thrombocytopenia—complicated by pulmonary infection, Aspergillus fumigatus infection, and influenza A. Following targeted anti-infective therapy at Sunshine Fusion Hospital, the patient’s condition improved, allowing discharge. In February 2025, due to further disease progression, the patient was readmitted and treated with two cycles of polatuzumab vedotin combined with rituximab and bendamustine (Pola+BR). On April 11, 2025, the patient succumbed to acute respiratory failure secondary to a pulmonary infection (**Figure 1C**).

## Discussion

This case report describes a rare clinical scenario in which diffuse large B-cell lymphoma (DLBCL) was diagnosed in a patient with gastric adenocarcinoma during postoperative treatment, including immune checkpoint inhibitor (ICI) therapy. While ICIs have shown significant efficacy in various malignancies, their increasing use has been accompanied by the recognition of rare or unexpected immune-associated events, particularly in less common oncological settings, as noted in a recent systematic review of ICIs in rare tumors (5). Hematologic complications, including sporadic reports of lymphoma occurring temporally with ICI exposure, have raised growing attention (6, 7).

In the present patient, pleural effusion developed during postoperative treatment with FOLFOX chemotherapy combined with the PD-1 inhibitor sintilimab. Cytological examination of the pleural fluid revealed atypical lymphoid cells, with occasional Epstein–Barr virus (EBV)–encoded RNA (EBER)-positive cells identified. These findings suggest the presence of immune perturbation during therapy; however, they do not, in isolation, establish a definitive diagnosis of lymphoma at that time. Immunophenotyping, flow cytometry, and clonality studies were not performed on the pleural effusion specimen, precluding a definitive distinction between a reactive lymphoid process and an evolving lymphoproliferative disorder. Given the effusion-driven presentation, the differential diagnosis should have explicitly included HHV-8–associated primary effusion lymphoma (PEL), HHV-8–negative, EBV-positive effusion-based large B-cell lymphoma in older adults, and other aggressive B-cell lymphomas presenting with serous effusions. Importantly, HIV testing was negative and HHV-8 status was not assessed, which limits precise classification according to WHO/ICC criteria. Early pleural fluid findings must therefore be interpreted with caution, and diagnostic uncertainty at that time must be acknowledged.

A major limitation of this case is the absence of comprehensive baseline systemic staging prior to the initiation of sintilimab therapy. Whole-body PET/CT or contrast-enhanced CT imaging performed before immunotherapy was not available for review. As a result, the possibility of a pre-existing occult or indolent lymphoproliferative disorder cannot be excluded. This limitation directly affects the interpretation of causality, raising the alternative possibility that immunotherapy and/or chemotherapy may have unmasked or accelerated the clinical manifestation of an underlying process, rather than inducing *de novo* lymphomagenesis. Notably, postoperative PET/CT identified a solitary FDG-avid lymph node, which was not biopsied and was initially interpreted as metastatic gastric adenocarcinoma. Retrospective reassessment suggests that this lesion may alternatively represent early lymphomatous involvement. The FDG uptake patterns of lymphoma and gastric cancer metastases may overlap, and in the absence of histopathological confirmation, definitive attribution of this hypermetabolic lymph node is not possible. The subsequent development of pathologically confirmed DLBCL supports the possibility that this PET/CT abnormality reflected an early or subclinical lymphoproliferative process.

Gastric cancer, with approximately 90% of cases being gastric adenocarcinoma (8), is common worldwide, while DLBCL arises from complex dysregulation of B-cell development and differentiation (9). Genetic alterations involving MYC, BCL2, and BCL6 (10–12), as well as aberrant activation of signaling pathways such as NF-κB and PI3K/Akt (13), are critical in lymphomagenesis. Based on existing literature, we propose several hypothetical mechanisms through which PD-1 blockade–associated immune modulation could contribute to aberrant B-cell activation in susceptible individuals (**Figure 1D**). These mechanisms are speculative and are presented to illustrate biological plausibility rather than to imply causation based on this single case.

Secondary DLBCL arising in patients with gastric cancer appears to be uncommon. Immune checkpoint inhibitors enhance T-cell activity by disrupting PD-1/PD-L1 interactions, but in some individuals, this immune activation may be accompanied by dysregulated immune responses (14). Cytokines such as IL-21, IL-4, IL-10, IFN-γ, and TNF-α, which are influenced by T-cell activation, may indirectly affect B-cell proliferation and survival (15–18). In addition, PD-1 signaling plays a role in regulating B-cell homeostasis and antibody quality, and its blockade may perturb these processes (19). Altered follicular helper T-cell (Tfh) localization and function under PD-1 inhibition may further influence B-cell activation (20).

However, chemotherapy-related immune perturbation represents a significant alternative explanation. Oxaliplatin- and fluoropyrimidine-based regimens can induce lymphodepletion, alter antigen presentation, and disrupt immune homeostasis during immune reconstitution (21). Such changes may transiently create a permissive environment for lymphoproliferation, independent of or synergistic with immune checkpoint inhibition. Therefore, the emergence of DLBCL in this patient cannot be attributed solely to PD-1 blockade.

EBV-associated lymphoproliferative processes constitute another plausible explanation. EBV plays a well-established role in the pathogenesis of certain lymphoid malignancies (22).Although preoperative gastric biopsy specimens were negative for EBER, the detection of occasional EBER-positive cells in pleural effusion cytology may reflect EBV reactivation, infected bystander cells, or low-level EBV-associated lymphoproliferation. In the absence of comprehensive EBV evaluation of diagnostic lymphoma tissue, including EBER *in situ* hybridization on tissue or cell-block specimens and/or EBV DNA analysis, EBV-related mechanisms cannot be definitively excluded (23).

In summary, this case illustrates a temporal association between PD-1 inhibitor–based therapy and the subsequent diagnosis of DLBCL in a patient with gastric adenocarcinoma. However, given the absence of baseline systemic staging, overlapping PET/CT findings, early diagnostic uncertainty in pleural effusion cytology, and the potential contributions of chemotherapy-related immune alteration and EBV-associated processes, a direct causal relationship cannot be established. This report underscores the importance of comprehensive baseline evaluation and careful longitudinal assessment in patients receiving immunotherapy, particularly when atypical lymphoid findings emerge during treatment.

### Clinical take-home messages

1. Comprehensive Baseline Evaluation Is Critical: Patients undergoing immune checkpoint inhibitor therapy, particularly in complex oncologic scenarios, should receive thorough baseline staging (e.g., PET/CT, histopathology) to enable more accurate monitoring of treatment-related immune events and to distinguish *de novo* lymphomagenesis from reactivation or progression of pre-existing conditions.
2. Immunotherapy May Unmask Underlying Lymphoproliferative Disorders: The use of PD-1 inhibitors like sintilimab can lead to immune modulation that may unmask subclinical or indolent lymphoproliferative processes, which may be exacerbated by chemotherapy, complicating the interpretation of post-treatment findings.
3. Interpreting Atypical Lymphoid Findings Requires Caution: Early cytological findings, such as the presence of atypical lymphoid cells in pleural effusions, should be interpreted with caution. Diagnostic uncertainty can be high without confirmatory tests (e.g., flow cytometry, clonality studies, EBV analysis) and can result in misinterpretation of immune-associated events as lymphoma.
4. Potential Role of EBV in Lymphoproliferation: Although EBV was not detected preoperatively in gastric tissue, reactivation of EBV during immunotherapy could contribute to the development of EBV-associated lymphoproliferative disorders, necessitating careful monitoring of EBV markers during treatment.
5. Multidisciplinary Approach Is Essential: Given the complex interactions between immunotherapy, chemotherapy, and viral reactivation in cancer treatment, a multidisciplinary approach involving oncologists, immunologists, and pathologists is essential for accurate diagnosis and management of immune-related adverse events.

## Conclusion

This case highlights the importance of vigilant monitoring of immune responses in patients treated with immune checkpoint inhibitors. In particular, the development of pleural effusion, new-onset lymphadenopathy, or systemic symptoms such as chest tightness should raise clinical suspicion for secondary lymphoma. Multidisciplinary collaboration, integrating pathological and immunological assessments, is essential for the timely recognition and management of immune-related adverse events. Although immune checkpoint inhibitors have demonstrated substantial therapeutic benefit in oncology, the potential risk of immune-mediated complications, including secondary diffuse large B-cell lymphoma (DLBCL), warrants careful and sustained attention.

## Data Availability

The original contributions presented in the study are included in the article/supplementary material. Further inquiries can be directed to the corresponding author/s.
